# A Case of Toxic Epidermal Necrolysis-Like Dermatosis Associated With Contact Exposure to a Flurbiprofen Plaster

**DOI:** 10.7759/cureus.104009

**Published:** 2026-02-21

**Authors:** Joohyung Youh, Yasuyuki Yamaguchi, Yukiko Nomura

**Affiliations:** 1 Department of Dermatology, Japan Railways (JR) Sapporo Hospital, Sapporo, JPN; 2 Department of Dermatology, Kokkakōmuin Kyōsai Kumiai Rengōkai (KKR) Sapporo Medical Center, Sapporo, JPN

**Keywords:** contact dermatitis, flurbiprofen plaster, severe cutaneous adverse reaction, ten-like dermatitis, topical nsaid, toxic epidermal necrolysis

## Abstract

Topical nonsteroidal anti-inflammatory drug (NSAID) formulations are widely used for the management of chronic musculoskeletal pain and are generally well tolerated. Severe cutaneous adverse reactions to topical agents are rare. We report a case of toxic epidermal necrolysis (TEN)-like dermatitis arising from contact dermatitis induced by a flurbiprofen plaster in an 81-year-old man. The patient developed erythroderma, extensive erosions, and systemic manifestations after two days of plaster application to the lumbar region.

The clinical presentation initially suggested TEN; however, histopathologic examination revealed marked dermal lymphocytic infiltration without keratinocyte necrosis, a finding inconsistent with true TEN. Direct and indirect immunofluorescence studies and serologic testing for autoimmune bullous and connective tissue diseases were negative.

The patient was treated with methylprednisolone pulse therapy followed by systemic corticosteroids and supportive care, achieving complete re-epithelialization after two months. This case demonstrates that severe contact dermatitis caused by topical NSAIDs can clinically mimic TEN and underscores the critical importance of histopathologic evaluation in establishing an accurate diagnosis.

## Introduction

Toxic epidermal necrolysis (TEN) is a life-threatening mucocutaneous adverse reaction most commonly triggered by systemic medications and characterized by widespread epidermal necrolysis, mucosal involvement, and systemic complications [1,2]. Because multiple dermatoses may present with TEN-like epidermal detachment, early clinicopathologic correlation and systematic exclusion of mimickers are essential [3,4]. Delayed or incorrect diagnosis leads to inappropriate prognostic counseling, suboptimal treatment selection, and unnecessary escalation to high-risk immunosuppressive regimens.

TEN mimickers include severe contact dermatitis, generalized bullous fixed drug eruption, autoimmune blistering diseases, acute graft-versus-host disease, phototoxic reactions, acute generalized exanthematous pustulosis, and pustular psoriasis. Histopathologic examination is critical for distinguishing cytotoxic from inflammatory etiologies [3-6]. This distinction is clinically essential because prognosis, pathophysiology, and management differ substantially. TEN has 20%-50% mortality depending on TBSA involvement [7], whereas severe contact dermatitis with secondary generalization typically has a favorable prognosis with supportive care and corticosteroids [8,9].

Flurbiprofen, a propionic acid derivative nonsteroidal anti-inflammatory drug (NSAID), is available in oral and topical plaster formulations. While Stevens-Johnson syndrome (SJS) from oral flurbiprofen has been reported [10], TEN-like reactions from topical plaster application remain exceptionally rare. Allergic contact dermatitis to topical NSAIDs is well documented, with ketoprofen, diclofenac, and flurbiprofen recognized as sensitizers [11,12]. However, progression from localized contact dermatitis to TEN-like generalized epidermal detachment is exceedingly uncommon. The broader literature on contact-triggered TEN-like eruptions predominantly involves industrial allergens, acrylate compounds, or chemical sensitizers rather than pharmaceutical topical preparations [13-15]. Clinical studies of S-flurbiprofen plasters report localized application-site dermatitis in 1%-3% of patients, but severe generalized TEN-like reactions have not been systematically characterized [16].

We describe a patient who developed a TEN-like dermatosis following application of a flurbiprofen-containing medicated plaster. Histopathologic findings excluded drug-induced TEN and instead supported TEN-like dermatosis associated with contact exposure to a flurbiprofen plaster, presenting with extensive epidermal detachment. This case highlights important diagnostic and prognostic pitfalls when TEN mimickers are not promptly recognized and underscores the limitations of applying TEN-derived mortality prediction tools, such as SCORTEN, to non-cytotoxic inflammatory detachment syndromes, particularly within the emerging clinicopathologic framework of contact exposure-associated TEN-like dermatoses.

## Case presentation

An 81-year-old man with no significant dermatologic history presented with severe pruritus, erythroderma, and erosions after applying a flurbiprofen-containing medicated plaster to the lumbar region for chronic back pain. The plaster contained flurbiprofen 40 mg and the following excipients: crotamiton, isopropyl myristate, glycerol, titanium dioxide, carmellose sodium, talc, a pH-adjusting agent, polysorbate 80, sorbitan sesquioleate, partially neutralized polyacrylic acid, levomenthol, and dried aluminum hydroxide gel.

**Figure 1:**
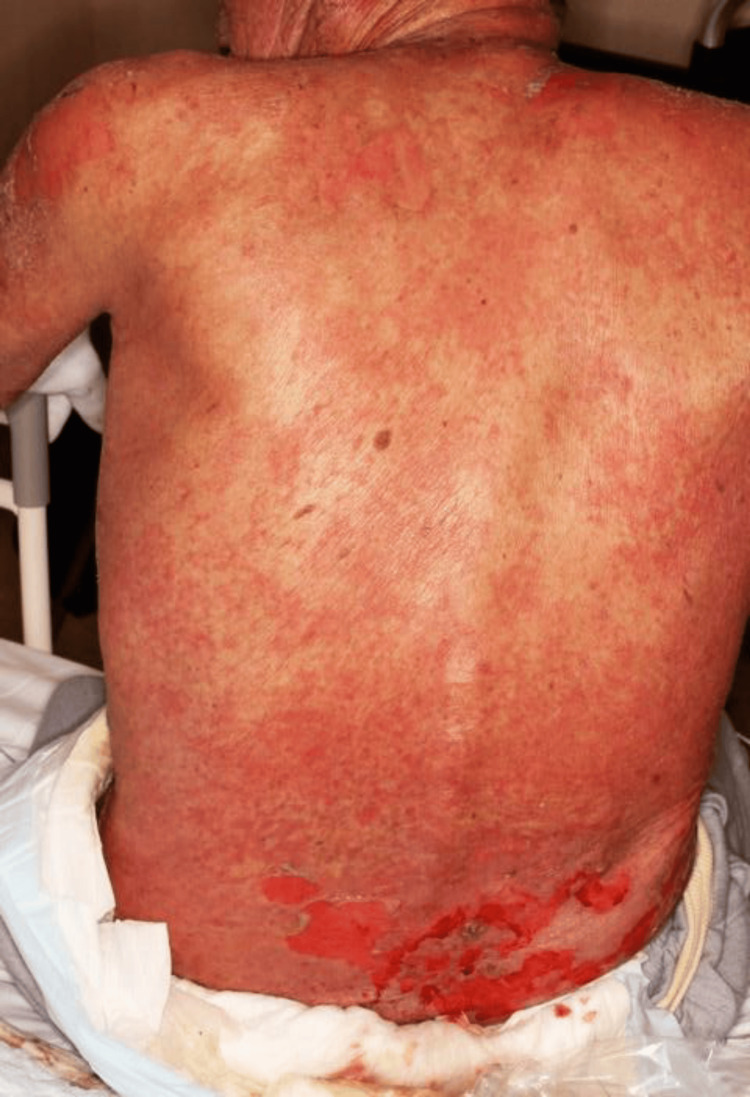
Exposure-site erosive dermatitis at initial presentation Multiple erosions and diffuse erythema at presentation on the first dermatology visit (approximately day 2 after initial plaster application), maximal at the plaster application site. The distribution demonstrates early exposure-site predominance.

Pruritus began within hours of first application (day 0). He presented to our dermatology clinic on day 2 with erythema, edema, and multiple superficial erosions, most pronounced at the plaster application site (Figure 1).

Despite medical advice, he continued plaster application through day 1, discontinuing on the morning of day 2 (Table 1).

**Table 1:** Clinical timeline of TEN-like epidermal detachment after flurbiprofen plaster application Day 0 indicates the first application of the flurbiprofen plaster. TBSA: total body surface area, ICU: intensive care unit, CRP: C-reactive protein, DIF/IIF: direct/indirect immunofluorescence, TEN: toxic epidermal necrolysis

Relative day*	Key clinical findings	Key investigations	Management/outcome
0	First flurbiprofen plaster applied to the lumbar area; pruritus began soon after	Not performed	Continued plaster use for two days
2	Dermatology visit: erythema and erosions maximal at the application site (Figure 1)	Not performed	Plaster discontinued
6	Rapid generalization; ~50% TBSA erosions/denudation with fever; oliguria, tachycardia, hypotension; no oral/ocular/genital mucosal involvement (Figure 2)	CRP: 25.95 mg/dL, albumin: 2.3 g/dL, renal/hepatic function preserved	ICU admission for monitoring and supportive care
6	Diagnostic evaluation during ICU admission	Skin biopsy: dense dermal lymphocytic infiltrate without keratinocyte necrosis (Figure 3); DIF/IIF negative; serologies for autoimmune bullous/connective tissue diseases negative	TEN less likely; severe contact dermatitis with TEN-like detachment favored
6-8	Systemic treatment phase	Not applicable	Methylprednisolone 1 g/day for three days
9-~60	Gradual re-epithelialization	Not applicable	Oral prednisolone 1 mg/kg/day tapered with wound care; complete re-epithelialization by ~2 months

**Figure 2:**
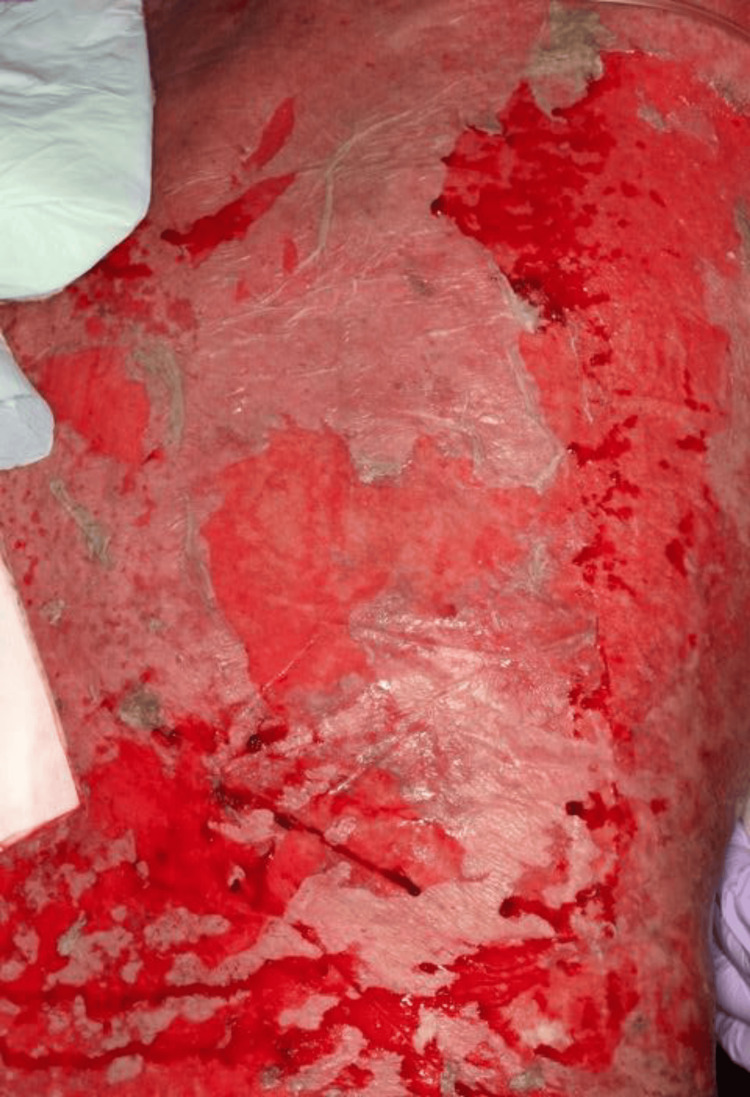
TEN-like widespread epidermal detachment Widespread TEN-like erosions and epidermal detachment (approximately day 6), involving approximately 50% TBSA, without oral, ocular, or genital mucosal involvement. Despite extensive epidermal loss, denudation remained most severe at the original plaster application site. TBSA: total body surface area, TEN: toxic epidermal necrolysis

**Figure 3:**
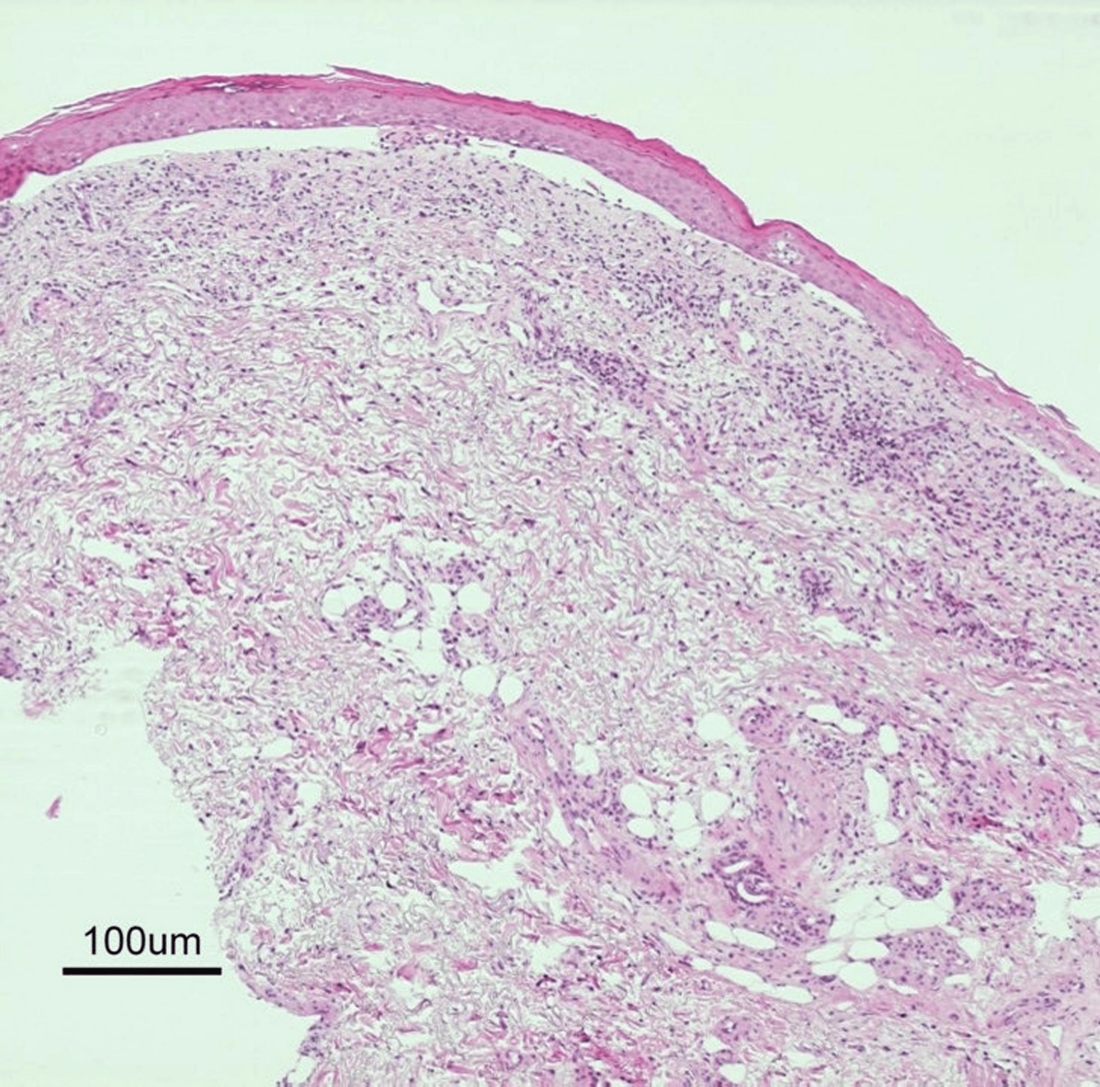
Low-magnification histopathology showing an inflammatory dermatitis pattern Histopathology (hematoxylin and eosin stain, ×40) demonstrating an inflammatory dermatitis pattern characterized by dense superficial and mid-dermal lymphocytic infiltration with focal spongiosis. There was no confluent epidermal necrosis, no full-thickness keratinocyte necrosis, and no subepidermal blister formation. Satellite cell necrosis and extensive interface change are absent, findings inconsistent with cytotoxic drug-induced toxic epidermal necrolysis and supportive of TEN-like dermatosis associated with contact exposure. TEN: toxic epidermal necrolysis

He denied a history of atopic dermatitis, contact allergy, or prior adverse drug reactions. Detailed medication history revealed no newly initiated systemic medications, over-the-counter drugs, herbal preparations, or additional topical agents within five years. He was not taking medications known to cause TEN. There was no recent infectious illness, vaccination, or travel.

On day 6, he developed rapidly progressive, widespread erosions and erythroderma involving approximately 50% of the total body surface area (TBSA), with the most severe denudation remaining at the original plaster application site (Figure 2), consistent with exposure-site-predominant TEN-like dermatosis associated with contact exposure.

**Figure 4:**
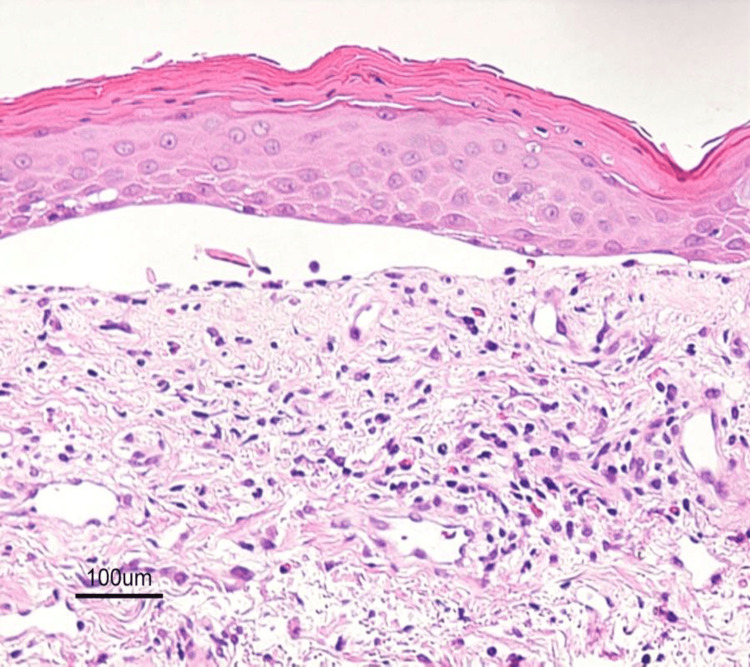
High-magnification histopathology supporting contact dermatitis Higher-magnification histopathology (hematoxylin and eosin stain, ×400) demonstrated dense dermal lymphocytic infiltration with scattered eosinophils and preserved epidermal architecture. Direct and indirect immunofluorescence studies were negative. Serologic evaluation for autoimmune blistering disorders, including BP180, BP230, and desmoglein 1 and 3 antibodies, was also negative. Histopathologic examination does not demonstrate epidermal necrosis or full-thickness keratinocyte death sufficient to meet the diagnostic criteria for TEN, supporting a diagnosis of TEN-like dermatosis associated with contact exposure rather than true TEN. TEN: toxic epidermal necrolysis

The Nikolsky sign was positive. However, no necrotic keratinocytes were observed in a biopsy specimen obtained from a Nikolsky-positive area (Figure 3 and Figure 4).

TBSA involvement was estimated using the Lund-Browder method. He was febrile (38°C), oliguric (<400 mL/24 hours), tachycardic (approximately 110 bpm), and hypotensive (approximately 80/60 mmHg), prompting intensive care unit (ICU) admission for hemodynamic monitoring and supportive care. Mucosal examination revealed no oral, ocular, or genital involvement.

Laboratory results on day 6 showed a white blood cell count of 4.3 × 10³/µL (reference: 4-10 × 10³/µL), C-reactive protein of 25.95 mg/dL (reference: <0.5 mg/dL), and serum albumin of 2.3 g/dL (reference: 3.5-5.0 g/dL). Renal function (blood urea nitrogen: 18 mg/dL, creatinine: 0.9 mg/dL) and hepatic function (aspartate aminotransferase (AST): 28 U/L, alanine aminotransferase (ALT): 22 U/L) were preserved. Serum glucose was 102 mg/dL and bicarbonate 24 mEq/L. Retrospective SCORTEN calculation yielded a score of 3, corresponding to a predicted mortality of 35.3% in validated toxic epidermal necrolysis (TEN) cohorts [7]. However, such estimates, derived from cytotoxic drug-induced TEN, may not apply to inflammatory mimickers, including TEN-like dermatosis associated with contact exposure.

A skin biopsy obtained on day 6 from the erythematous margin adjacent to a fresh trunk erosion demonstrated dense superficial and mid-dermal lymphocytic infiltration with focal spongiosis and mild epidermal acanthosis (Figure 3 and Figure 4). Critically, there was no confluent epidermal necrosis, no full-thickness keratinocyte apoptosis, and no subepidermal blister formation. Satellite cell necrosis and extensive vacuolar interface change, hallmarks of drug-induced TEN, were absent. Direct immunofluorescence of perilesional skin was negative for IgG, IgA, IgM, C3, and fibrinogen. Indirect immunofluorescence on monkey esophagus and salt-split skin substrates was negative. Serologic testing for autoimmune blistering disorders, including ELISA for BP180, BP230, desmoglein 1, and desmoglein 3 antibodies, was negative, supporting an inflammatory contact exposure-associated TEN-like dermatosis rather than cytotoxic epidermal necrolysis.

Given extensive detachment (50% TBSA) and hemodynamic instability, empiric intravenous methylprednisolone 1,000 mg daily was initiated on day 6 pending histopathologic confirmation. Following biopsy results on day 8, the diagnosis was revised to severe exposure-site contact dermatitis with secondary generalization, within the clinicopathologic spectrum of TEN-like dermatosis associated with contact exposure to a flurbiprofen plaster. He received methylprednisolone pulse therapy 1,000 mg daily for three days (days 6-8), followed by oral prednisolone 60 mg daily (~1 mg/kg/day), tapered by 10 mg every five days over four weeks. Supportive care included intravenous fluid resuscitation, vasopressor support (discontinued day 8), continuous hemodynamic monitoring, silver sulfadiazine-impregnated foam dressings, and close monitoring for secondary bacterial infection. Complete re-epithelialization was achieved by eight weeks (day 56). He was discharged on day 18 with oral prednisolone taper and outpatient follow-up. No recurrence occurred during the six-month follow-up.

## Discussion

TEN most commonly results from systemic medication exposure, particularly allopurinol, sulfonamide antibiotics, anticonvulsants (carbamazepine, phenytoin, and lamotrigine), and oxicam NSAIDs [2,17]. However, several inflammatory conditions may mimic TEN with extensive epidermal detachment [3,4].

The defining diagnostic clue was anatomic distribution: disease severity was maximal at the topical plaster application site throughout the clinical course. While generalization to 50% TBSA occurred, the lower back demonstrated the most severe denudation and the slowest re-epithelialization. This pattern is atypical for systemic drug-induced TEN, which produces symmetric and anatomically random epidermal detachment without preferential localization [2,17]. Clinically, the presentation most closely resembled TEN; however, there was insufficient histopathologic evidence to support a definitive diagnosis. We therefore considered the term “TEN-like dermatosis associated with contact exposure to a flurbiprofen plaster” to be the most appropriate descriptor of this patient’s cutaneous condition.

Severe contact dermatitis may generalize through distinct mechanisms. Autoeczematization (id reaction) involves immunologic spread via lymphatic dissemination of haptenized proteins or immune complexes, producing distant eczematous lesions morphologically similar to the primary site [8,9]. Systemic contact dermatitis results from hematogenous dissemination after percutaneous absorption of a contact allergen to which the patient was previously sensitized [8]. A third mechanism involves the Koebner phenomenon or irritant-driven barrier disruption facilitating secondary allergen penetration. In this case, rapid generalization within six days, systemic inflammatory symptoms, and absence of documented prior sensitization make classic systemic contact dermatitis less likely than a severe exposure-site inflammatory reaction with secondary immunologic propagation within the spectrum of TEN-like dermatosis associated with contact exposure.

Causality attribution warrants systematic analysis. Retrospective Naranjo Adverse Drug Reaction Probability Scale yielded score 6 (probable causality), based on the following: (1) temporal correlation with first exposure, (2) clinical improvement following plaster withdrawal, (3) anatomic correspondence between maximal severity and application site, and (4) absence of alternative systemic drug triggers. However, the multi-ingredient plaster composition (flurbiprofen plus 11 excipients, including known sensitizers: crotamiton, levomenthol, and partially neutralized polyacrylic acid) precludes definitive attribution to flurbiprofen alone. Patch testing was not performed due to clinical instability during acute illness and patient reluctance during convalescence. Therefore, causality is attributed to the plaster system as a whole as the contact exposure precipitating the TEN-like dermatosis phenotype.

Contact-triggered TEN-like presentations have been described with industrial dendrimers, acrylate compounds, and ultraviolet-cured inks [13-15]. Topical NSAIDs are recognized causes of allergic and photoallergic contact dermatitis, with diclofenac, ketoprofen, and piroxicam most frequently implicated [11,12]. However, TEN-like generalized detachment following topical NSAID application remains exceedingly rare. To our knowledge, this represents the first detailed case report of TEN-like dermatosis associated with contact exposure to a flurbiprofen plaster, with comprehensive histopathologic documentation excluding drug-induced TEN.

Histopathology provided decisive diagnostic clarity. Classic drug-induced TEN demonstrates confluent full-thickness epidermal necrosis with widespread keratinocyte apoptosis, minimal dermal inflammation (“quiet” dermis), and subepidermal blister formation [17]. In contrast, inflammatory mimickers such as severe contact dermatitis show preserved epidermal viability, prominent dermal inflammatory infiltrates, spongiotic change, and absence of confluent pan-epidermal necrosis. This clinicopathologic discordance (extensive clinical detachment with histologic preservation of epidermal architecture) is a critical diagnostic red flag prompting reconsideration of TEN diagnosis and systematic evaluation for mimickers [3,4]. This case demonstrated dense superficial and mid-dermal lymphocytic infiltration with focal spongiosis, supporting a contact exposure-associated TEN-like dermatosis rather than cytotoxic drug-induced epidermal necrolysis.

Additional distinguishing features included absence of satellite cell necrosis, minimal vacuolar interface change, and absence of subepidermal blister formation. These findings, combined with negative direct and indirect immunofluorescence, excluded autoimmune blistering diseases and supported an inflammatory contact exposure-driven TEN-like dermatosis.

Integrating distributional morphology (exposure-site predominance), mucosal assessment (complete sparing despite 50% TBSA detachment), and histopathologic injury pattern (inflammatory infiltrate without keratinocyte necrosis), we propose a practical bedside diagnostic triad for identifying TEN mimickers from topical exposures within the conceptual framework of TEN-like dermatosis associated with contact exposure: absence of mucosal involvement despite extensive TBSA detachment, maximal severity at a discrete exposure site during the early disease course, and histopathologic absence of confluent full-thickness keratinocyte necrosis.

This framework is conceptual and intended to guide early clinical reasoning rather than serve as a validated diagnostic rule. When all three criteria are met, clinicians should strongly consider contact exposure-associated TEN-like dermatosis or related inflammatory detachment syndromes rather than drug-induced TEN. Prospective validation in larger case series is needed.

Favorable outcome despite extensive detachment and SCORTEN score predicting 35% mortality suggests prognostic indices derived from cytotoxic drug-induced epidermal necrolysis cohorts substantially overestimate mortality risk in inflammatory detachment syndromes. SCORTEN was developed and validated in cohorts of cytotoxic drug-induced SJS/TEN confirmed by histopathologic demonstration of keratinocyte necrosis [7]. Applying SCORTEN to TEN mimickers, where underlying pathophysiology is fundamentally different, may lead to inappropriate prognostic counseling and unnecessary escalation to high-risk immunosuppressive therapies such as intravenous immunoglobulin, cyclosporine, or anti-TNF biologics [18]. While corticosteroids remain controversial in true TEN due to infection concerns and conflicting efficacy data [18], they may be appropriate first-line therapy in inflammatory mimickers, including TEN-like dermatosis associated with contact exposure, where immune-mediated inflammation predominates over cytotoxic keratinocyte death.

Alternative differential diagnoses were systematically excluded through clinicopathologic correlation (Table 2).

**Table 2:** Differential diagnosis of TEN-like widespread epidermal detachment and case-based interpretation SJS: Stevens-Johnson syndrome, TEN: toxic epidermal necrolysis, GBFDE: generalized bullous fixed drug eruption, BP: bullous pemphigoid, PV: pemphigus vulgaris, DIF/IIF: direct/indirect immunofluorescence, BMZ: basement membrane zone, SSSS: staphylococcal scalded skin syndrome

Differential diagnosis	Typical discriminators (bedside + key tests)	Findings in this patient → interpretation
SJS/TEN (drug-induced)	Often severe skin pain; mucosal involvement common; recent high-risk systemic drug exposure; biopsy shows widespread keratinocyte necrosis (often full-thickness)	No new systemic drug exposure; no mucosal involvement; biopsy showed no keratinocyte necrosis → true TEN unlikely
TEN-like dermatosis associated with contact exposure	Maximal severity at exposure site (early); prominent pruritus; may generalize (autoeczematization/systemic contact dermatitis spectrum); biopsy shows inflammatory dermatitis with absent/limited necrosis	Exposure-site maximal involvement from day 2; marked pruritus; temporal link to topical plaster; biopsy: dense dermal lymphocytic infiltrate without necrosis →findings most consistent with TEN-like dermatosis associated with contact exposure
GBFDE	Often recurrent with re-exposure; well-demarcated dusky plaques; biopsy shows interface dermatitis with necrotic keratinocytes and pigment incontinence	No prior similar episodes; no re-exposure pattern; onset/maximal severity at plaster site rather than classic fixed lesions → less likely
Autoimmune blistering disease (BP/PV/linear IgA, etc.)	DIF typically positive (e.g., linear IgG/C3 at BMZ in BP; intercellular IgG in PV); IIF/ELISA supportive	DIF negative; IIF negative; serologies negative → unlikely
SSSS	Typically children; adults usually immunocompromised; mucosa usually spared; biopsy shows superficial intraepidermal split (granular layer)	Clinical context and reported histopathology not supportive of superficial split typical of SSSS → unlikely

Generalized bullous fixed drug eruption typically presents with well-demarcated dusky erythematous plaques recurring at the same anatomic sites upon re-exposure, often with post-inflammatory hyperpigmentation [19]. Histopathology shows interface dermatitis with necrotic keratinocytes and prominent pigment incontinence, features absent in this case. Autoimmune blistering diseases were excluded by negative direct and indirect immunofluorescence and the absence of circulating autoantibodies. Staphylococcal scalded skin syndrome was unlikely given the patient’s age, absence of immunocompromise, and histopathologic findings inconsistent with superficial intraepidermal split typical of this condition. Acute generalized exanthematous pustulosis was excluded due to the absence of non-follicular sterile pustules and predominantly lymphocytic rather than neutrophilic infiltrate.

Limitations include the following: (1) absence of patch testing to identify specific causative component within multi-ingredient plaster formulation, (2) single-site biopsy sampling, (3) lack of re-exposure data to confirm causality, and (4) inherent constraints of single-case observational design precluding generalizability. Future prospective studies incorporating patch testing protocols and multi-site biopsies are needed to better characterize TEN-like dermatosis associated with contact exposure as a rare adverse reaction pattern.

## Conclusions

Severe exposure-site contact dermatitis induced by topical NSAID-containing medicated plasters may rarely present with TEN-like erythroderma, widespread epidermal detachment, and systemic inflammatory manifestations, mimicking drug-induced toxic epidermal necrolysis. Within this spectrum, TEN-like dermatosis associated with contact exposure to a flurbiprofen plaster represents a distinct clinicopathologic entity characterized by exposure-site predominance and inflammatory rather than cytotoxic epidermal injury. Recognition of this clinicopathologic pattern may prevent misdiagnosis, inappropriate application of TEN mortality prediction scores, and unnecessary escalation to high-risk immunosuppressive regimens. Early biopsy from erythematous advancing edge, meticulous exposure-site anatomic mapping, and careful clinicopathologic correlation are essential when managing patients with TEN-like eruptions. Clinicians should maintain a high index of suspicion for TEN mimickers, particularly when exposure-site predominance is evident and mucosal involvement is absent despite extensive body surface area detachment.

## Appendices

Learning points

Three red flags suggest TEN mimickers: (1) complete mucosal sparing despite extensive skin detachment, (2) maximal severity localized to a discrete exposure site, and (3) intense pruritus rather than severe pain. These features warrant early diagnostic biopsy before initiating systemic immunosuppression, particularly when considering the possibility of TEN-like dermatosis associated with contact exposure.

Histopathology is decisive for diagnosis and treatment. Dense dermal lymphocytic infiltration with preserved epidermal architecture indicates an inflammatory mimicker, whereas confluent full-thickness keratinocyte necrosis confirms true TEN. This distinction fundamentally alters prognosis and therapy; corticosteroids may be first-line for inflammatory mimickers, including contact exposure-associated TEN-like dermatosis, but remain controversial in true TEN.

SCORTEN substantially overestimates mortality in inflammatory detachment syndromes. Do not quote TEN mortality predictions until histopathology confirms keratinocyte necrosis. Avoid reflexive escalation to high-risk therapies (IVIG, cyclosporine, and anti-TNF biologics) when histology shows inflammatory mimicry; corticosteroids with supportive care may suffice, particularly in TEN-like dermatosis associated with contact exposure where cytotoxic epidermal injury is absent.

Systematically evaluate topical exposures in exposure-site predominant eruptions. Topical NSAID plasters and medicated adhesive systems can cause severe generalized reactions. Obtain detailed exposure history, including over-the-counter products, complementary medicines, and occupational contactants. When patch testing is not feasible, attribute causality to the complete product formulation rather than isolated ingredients, as the composite exposure may underlie TEN-like dermatosis associated with contact exposure.

## References

[1] Frantz R, Huang S, Are A, Motaparthi K (2021). Stevens-Johnson syndrome and toxic epidermal necrolysis: a review of diagnosis and management. Medicina (Kaunas).

[2] Schwartz RA, McDonough PH, Lee BW (2013). Toxic epidermal necrolysis: part I. Introduction, history, classification, clinical features, systemic manifestations, etiology, and immunopathogenesis. J Am Acad Dermatol.

[3] Creamer D, Walsh SA, Dziewulski P (2016). U.K. guidelines for the management of Stevens-Johnson syndrome/toxic epidermal necrolysis in adults 2016. Br J Dermatol.

[4] Weinkle A, Pettit C, Jani A (2019). Distinguishing Stevens-Johnson syndrome/toxic epidermal necrolysis from clinical mimickers during inpatient dermatologic consultation-a retrospective chart review. J Am Acad Dermatol.

[5] Salah E (2023). TEN mimics: classification and practical approach to toxic epidermal necrolysis-like dermatoses. Indian J Dermatol Venereol Leprol.

[6] Shah H, Parisi R, Mukherjee E, Phillips EJ, Dodiuk-Gad RP (2024). Update on Stevens-Johnson syndrome and toxic epidermal necrolysis: diagnosis and management. Am J Clin Dermatol.

[7] Bastuji-Garin S, Fouchard N, Bertocchi M, Roujeau JC, Revuz J, Wolkenstein P (2000). SCORTEN: a severity-of-illness score for toxic epidermal necrolysis. J Invest Dermatol.

[8] Aquino M, Rosner G (2019). Systemic contact dermatitis. Clin Rev Allergy Immunol.

[9] Scheinman PL, Vocanson M, Thyssen JP (2021). Contact dermatitis. Nat Rev Dis Primers.

[10] Khan AA, Rashid F, Khan FU, Tahmina T, Amin S, Anand A (2022). Diagnosis and treatment of flurbiprofen-induced Stevens-Johnson syndrome: a rare case report. Clin Case Rep.

[11] Gulin SJ, Chiriac A (2016). Diclofenac-induced allergic contact dermatitis: a series of four patients. Drug Saf Case Rep.

[12] Ophaswongse S, Maibach H (1993). Topical nonsteroidal antiinflammatory drugs: allergic and photoallergic contact dermatitis and phototoxicity. Contact Dermatitis.

[13] Toyama T, Matsuda H, Ishida I, Tani M, Kitaba S, Sano S, Katayama I (2008). A case of toxic epidermal necrolysis-like dermatitis evolving from contact dermatitis of the hands associated with exposure to dendrimers. Contact Dermatitis.

[14] Ido T, Kiyohara T, Takahashi H, Yamaguchi Y, Tani D, Kumakiri M (2012). Toxic epidermal necrolysis following allergic contact dermatitis caused by occupational exposure to ultraviolet-cured inks. Acta Derm Venereol.

[15] Jiang W, Guo T, Song M, Ma X, Zhang J (2024). A case of contact toxic epidermal necrolysis. Indian J Dermatol.

[16] Yataba I, Otsuka N, Matsushita I, Matsumoto H, Hoshino Y (2016). The long-term safety of S-flurbiprofen plaster for osteoarthritis patients: an open-label, 52-week study. Clin Drug Investig.

[17] Schwartz RA, McDonough PH, Lee BW (2013). Toxic epidermal necrolysis: part II. Prognosis, sequelae, diagnosis, differential diagnosis, prevention, and treatment. J Am Acad Dermatol.

[18] Jacobsen A, Olabi B, Langley A (2022). Systemic interventions for treatment of Stevens-Johnson syndrome (SJS), toxic epidermal necrolysis (TEN), and SJS/TEN overlap syndrome. Cochrane Database Syst Rev.

[19] Balta I, Simsek H, Simsek GG (2014). Flurbiprofen-induced generalized bullous fixed drug eruption. Hum Exp Toxicol.

